# Beewatching: A Project for Monitoring Bees through Photos

**DOI:** 10.3390/insects12090841

**Published:** 2021-09-18

**Authors:** Simone Flaminio, Rosa Ranalli, Laura Zavatta, Marta Galloni, Laura Bortolotti

**Affiliations:** 1 CREA Research Centre for Agriculture and Environment, Via di Corticella n. 133, 40128 Bologna, Italy; simone.flaminio@crea.gov.it (S.F.); rosa.ranalli@crea.gov.it (R.R.); laura.zavatta@crea.gov.it (L.Z.); 2 Department of Biological, Geological and Environmental Sciences, University of Bologna, Via Irnerio 42, 40126 Bologna, Italy; marta.galloni@unibo.it

**Keywords:** wild bees, citizen science, plant pollinator interactions, pollinators, pollination network

## Abstract

**Simple Summary:**

Citizen science is the involvement of the public in scientific research, through the collection of data by volunteer members (“citizen scientists”), usually as part of a collaborative project with professional scientists. Beewatching is a citizen science project started in spring 2018. The project target is to acquire information on wild bee species in Italy and to promote knowledge among citizens on the diversity of bees and their importance as pollinators. Moreover, an attempt was made to follow the distribution of two alien bee species present in Italy. During the first two years of the project, 269 users contributed with 1086 reports. Overall, 38 Apoidea genera and 190 plant genera were reported. Furthermore, we received 22 reports of the alien species *Megachile sculpturalis*. The increasing number of reports and the higher percentage of correct ones throughout the first period of project implementation, but also the ecological data acquired, undoubtfully represent positive outputs, demonstrating the success of Beewatching project.

**Abstract:**

Bees play a key role in natural and agro-ecosystems and their diversity is worldwide threatened by anthropogenic causes. Despite this, there is little awareness of the existence of the numerous species of wild bees, and the common name “bee” is very often exclusively associated with *Apis mellifera*. Our aim was to create a citizen science project in Italy with the following objectives: (a) raising awareness of the importance and diversity of bees, (b) obtaining data on the biology, ecology and distribution of Italian species, and (c) launching the monitoring of alien bees. The first step of the project was to create a website platform with a section containing informative datasheets of the wild bee families and of the most common bee genera present in Italy, a form to send reports of observed bees and an interactive map with all citizen’s reports. During the 2 years of the project 1086 reports were sent by 269 users, with 38 Apoidea genera reported on 190 plant genera; furthermore, 22 reports regarding the alien species *Megachile sculpturalis* arrived. The majority of bees (34 genera) were observed on spontaneous plants, including 115 genera native to Italy. Considering the increasing number of reports and data obtained in these first two years of the project, our objectives seem to be achieved. Future steps will be to outline the profile of beewatchers, to plan activities in a more targeted way, and also to start some sub-projects for conservation purposes.

## 1. Introduction

Citizen science (CS) is the involvement of the public in scientific research, through the collection of data by volunteer members (“citizen scientists”), usually as part of a collaborative project with professional scientists [1]. CS combines the objective of informing and training the public on certain topics and providing data on that topic to the scientists. The advantage of CS with respect to “traditional science” is to allow the collection of a large amount of data over wide areas, which would otherwise not be possible if conducted by paid professionals [2].

CS projects are increasingly implemented in many natural science fields, where collected data are used to monitor species and track populations, design distribution maps and make predictions about their spread. These data can be used also at policy level for defining conservation and management plans [3,4]. CS programs are traditionally used to survey and monitor native species, but recently their use in the detection and tracking of invasive alien species has increased, sometimes even as a part of a warning system [5].

Although the monitoring by citizen scientists is a good solution in cases of limited resources, since it allows large sampling at low costs, it implies a decrease in the accuracy of data collected, in comparison to those collected by experts. The most common error in a citizen science program is the misidentification of the species, which become very frequent in small organism like insects [6]. One way to reduce errors due to misidentification is to employ a “verified method”, in which all the observations collected or sent by citizens are verified by experts. This approach is more expensive since it requires verification by professional researchers, but it greatly increases the accuracy of the data collected [7].

To start an effective CS project, the dissemination of information is essential, in order to reach as many people as possible, who will subsequently act as citizen scientists. The use of Internet has broadened the chance for public outreach and engagement; Internet and social networks allow the spreading of real-time information and give to citizens the possibility to make immediate reports, inserting data and attaching photo. Citizen science implemented through photos and videos is quite widespread for naturalistic surveys, as it consents a non-destructive monitoring [8]. Thanks to the current technological development, more and more people have high quality cameras available on their mobile phone; furthermore, usually people with a naturalistic attitude dislike to kill animals or collect plants and they prefer to document their presence through photographs.

Due to their small size and species similarity, bees are particularly difficult to be identified by citizens. Moreover, very few identification tools are available on this group. Within the order Hymenoptera some bee and wasp species can be difficult to distinguish by non-professionals; people may even confuse insects belonging to different orders, as bees (Hymenoptera) with syrphids and bombyliids (Diptera) or with diurnal sphingids (Lepidoptera). For this reason, bee diversity studies are not commonly achieved by CS, but monitoring is rather entrusted to professionals, and species identification is reserved to specialized taxonomists [9].

The recognition of a bee species on a specific plant is a more easily achievable task for citizens. Some CS projects are focused on a single bee species, to be found on the plants, that preferentially visits (and pollinates) as the case of the squash bee *Eucera (Peponapis) pruinosa* (Say, 1837) on cucurbit flowers. Squash bees are ground nesting insects, and their monitoring can be useful for examining impacts of farm management practices on their nesting activity and pollinator efficacy, driving farming decisions [10]. Other CS projects are focused on a single plant species, used to monitor flower-visiting bees and quantify their pollination service; this approach implies a lower insect taxonomic diversity to be recognized, given that bees selectively choose their foraging plants [11].

Many of the projects that are based on the collection of bee photograph records focus on a single easy recognizable taxonomic group. The “Bumblebee Conservation Trust” [12] collects records of bees of the genus *Bombus* to promote the knowledge of British bumble bees and the conservation of their habitats; the Canadian “Bumble Bee Watch” [13] web sites collects reports of bumble bees photos verified by experts, in order to track and conserve North America’s bumble bees; “Blooms for Bees” was a three-year project aimed at gathering data on the floral preferences of British bumblebee species, using a smartphone app featuring a bumblebee identification guide [14]; the Hanamaru-Maruhana National Census was a Japanese program run from 2013 to 2015 and aimed at estimating the distributions of the six major species of bumblebees found in Japan [15]. Focusing on a single bee genus makes the validation process/taxonomic verification by experts an easier task, even at the specific level. In parallel, this approach allow the evaluation of citizens’ awareness (e.g., ability of citizen scientists to correctly identify the bee) [14] or even the use of data for scientific research [15].

Unlike CS projects that focus on a single genus, those aimed at monitoring whole bee or pollinator biodiversity in a given ecosystem often train people to identify bees at a taxonomic level higher than species, by pooling them into few easily identifiable groups. The California Pollinator Project [16] and the Great Pollinator Project [17] are American monitoring programmes aimed at collecting data on bee biodiversity and abundance in specific areas and to record changes in bee populations over time. In both projects bees are determined basing on immediate feature such as size, shape, colour, placement of pollen, etc. and pooled into broad morphological and functional categories (i.e., honey bees, bumble bees, large carpenter bees, hairy leg bees, green sweat bees, striped sweat bees, small dark bees, etc.). These surveys often involve photo documentation of the pollinators, which can allow researchers to verify observations and to investigate the effective ability of CS to describe pollinator communities [16,18]. Other photograph-based CS projects on bees, such as BeeWatch (https://beewatch.abdn.ac.uk/, accessed on 4 August 2021) or the Bees, Wasps, and Ants Recording Society (BWARS) (http://www.bwars.com, accessed on 4 August 2021) have sought to maintain the species-level recording through expert verification, with the result that many records are rejected as unidentifiable compared to the broad group approach (20).

The main limitation to CS projects on bees, especially when records are achieved by photographs, is the low taxonomic data quality. However, such data can still be very useful, if methods and protocols are developed taking into account the degree of inaccuracy of the data provided by volunteers compared to professionals. Kremen et al. [8] compared the data collected on pollinators (at the level of order or superfamily) by citizen scientists and by bee specialists in 17 sites: they found a positive correlation between the two datasets concerning pollinator abundance and richness among the sites, although citizen scientists observed only half of the bee groups collected by professional scientists.

Given the importance of bees for the maintenance of the ecosystem health [19], their current state of risk and consequent growing popularity among people, it is becoming increasingly urgent to develop monitoring systems that allow even non-professionals to return relevant data to scientists. Citizen science programs represent a significant step towards this goal, but they require that people become aware of the existence of many species of bees (often by “bee” they only mean the “honey bee”) and that scientists develop strategies to help citizens to recognize them, such as training session and simplified tools to determine taxonomic ranks higher than the species (e.g., genus or tribe). Citizen science projects often include trainings that educate volunteers to recognise bee groups, but they involve a limited number of people and reduce the possibility of carrying out monitoring in large areas. Possible solutions may be online trainings or tutorials and identification tools to be downloaded from a website.

Beewatching is a citizen science project started in spring 2018 from a collaboration between CREA (Council for Agricultural Research and Economics) and BiGeA Department (University of Bologna). The project target is to acquire information on wild bee species in Italy and to promote knowledge among citizens on the diversity of bees and their importance as pollinators. Moreover, an attempt was made to follow the distribution of two alien bee species present in Italy, *Megachile sculpturalis* Smith, 1853 [20] and *Megachile disjunctiformis* Cockerell, 1911 [21]. Beewatching is the first citizen science project in Italy and also one of the few performed worldwide that takes into consideration the entire group of Apoidea Apiformes. The name “Beewatching” was chosen as a derivation of the best known “birdwatching”, to describe the activity of observing bees in the environment, without capturing or killing them.

Here we report the results obtained after two years of project implementation, with the aim to verify how far the project has spread on the Italian territory, and assess the progresses achieved in terms of participation and recognition skills throughout this period.

## 2. Materials and Methods

### 2.1. Web Site

Considering that the main limitation in CS projects is people training and that in Italy the knowledge of the different species of bees among citizens is very limited, the first step of the project was to create a website platform (www.beewatching.it, accessed on 4 August 2021) providing a section with informative datasheets of the wild bee families and of the most common bee genera present in Italy. For each bee genus the description of the general appearance and life habits is included, together with pictures of some specimens, and a detailed description of the most common species, with photos of the male and female specimens. To date, the above-mentioned description is available online for 46 genera and about 150 species, but we plan to add more in the coming years. A section with tips on how to promote the presence of pollinators in private green areas (balconies, backyards, gardens), how to enhance their nesting and what plants to grow to better sustain them, is included.

The cores of the web site are the form to send reports of observed bees and the interactive map which shows all citizen’s reports. Instructions are given to the participants on how to photograph the bee, which shots are the best to allow identification and which additional information is useful to provide (e.g., a dimensional comparison).

The form to enter the reports requires the following data: name, surname, and e-mail address of the reporter; one or more photos of the bee; the date of the photographs; the indication of a size class: XS, Extremely Small (4–6 mm); S, Small (6–8 mm); M, Medium (8–13 mm); L, Large (13–16 mm); XL, Extra Large (>16 mm). Furthermore, the form includes two fields where the reporter can enter a tentative taxonomic identification of the bee and the visited flower. The geographical coordinates of the report (in decimal degrees, datum WGS84) can be entered by the user or be automatically generated by the form, according to the user’s position.

All the reports sent to the web site were verified by experts and any erroneous attribution on bee and plant species were corrected; once the photo was sent, the participants received an e-mail with a confirmation of receipt. After verification and validation, the photo with the correct data is recorded on the dataset and displayed on the website map: clicking on the single report, a pop up is open with the photo of the specimen and all record details.

### 2.2. Project Promotion

The project and the web sites were promoted over the two years through the seminars and courses organized by the researchers of the two institutions as part of their institutional and teaching activities.

Two informative videos were created describing the project and providing guidance on participation were distributed through “YouTube” channel.

For disseminating the project to a wider audience, a Facebook page was opened (https://www.facebook.com/BeewatchingIT, accessed on 4 August 2021), and posts dedicated to single species or genera are generated each week during the active season of pollinators (March–October) to increase the knowledge of the morphological characters useful for the recognition of species or genera to users. The bee species described in the posts in a certain period are those emerging in Italy just in that period, so that followers can recognize them in the environment during their “beewatching tours”.

This social platform is also used by some users to send reports through posts or private messages, but these reports were not counted among the project results, except those relating to alien species.

### 2.3. Project Implementation

**Figure 1:**
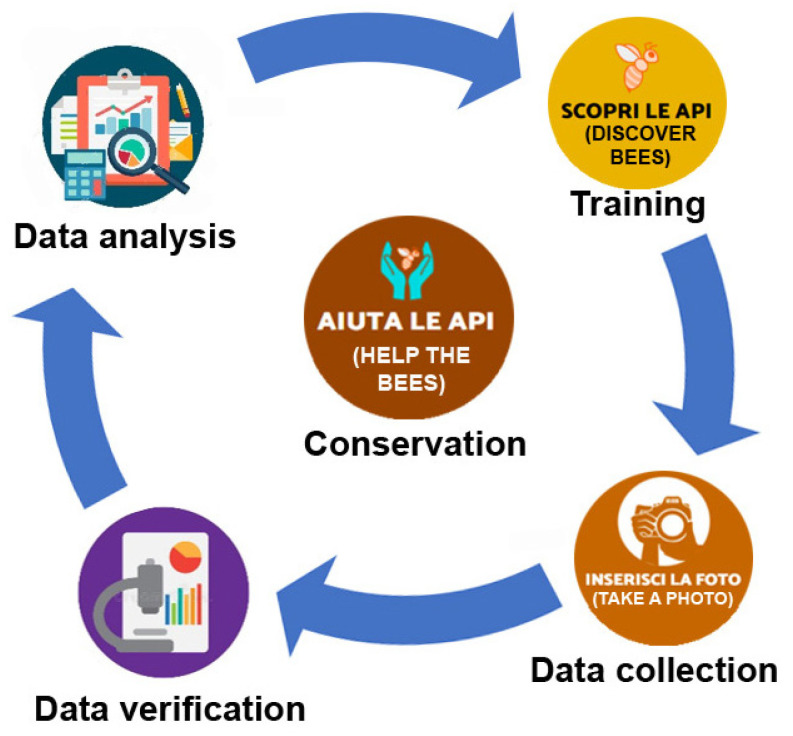
Circular process underlying the project: training, data collection, data verification, data analysis and new training; the final purpose is bee conservation.

The project followed a circular process through four different steps: (1) a “passive” training through the videos and the on-line datasheets on bee genera and species; (2) data collection through the photo sent by the beewatchers; (3) data verification by project experts; and (4) data analysis (Figure 1). New training actions can be planned from the considerations emerged by data analysis.

The final purpose of the whole project is bee conservation, achieved through the augmented knowledge and awareness of citizens on the importance of bees and their diversity.

### 2.4. Data Analysis

The data obtained in the first two years of activity of the project were classified according to the year of the report, the province and region of origin, the size (presumed) of the bee specimen, the genus and, when possible, the species of the photographed bee. Given the difficulty of identifying bees to the rank of species by the photograph, the identification by the users was considered correct when the assignment to the genus rank was correct. A $\chi^{2}$ was calculated to verify if the number of correct bee identification by users increases over the two years. In addition, the specimens were determined at the species rank by the project experts, to verify the possibility to identify them from the photographs.

The reports relating to alien bee species, both those received from the reports of the website and those from the Facebook page, were extracted to contribute to a national distribution map [22].

For each report, the visited plant (i.e., flower on which the bee was photographed) was also identified, either at specific or generic taxonomic level (when picture’s details did not allow to determine the plant species).

In order to standardise the information only the genus category was then considered in the analysis of data.

Each plant was assigned to the “spontaneous” or “cultivated” category, based on the photographed environment: if the plant was clearly growing in a backyard or a balcony, or in a flowerpot, then the category “cultivated” (C) was assigned. We included in this category also allochthonous ornamental plants, usually cultivars, and whose presence in nature is not documented. The Euro+Med PlantBase database [23] was used to verify the natural occurrence of these plants in Italy as spontaneous, allochthonous or invasive.

If, instead, the observation was made in a natural area, with no anthropic elements in the photograph, and the plant genus included autochthonous species, then the category “spontaneous” (S) was assigned.

Those genera including both spontaneous and cultivated species, such as *Calendula* and *Cyclamen*, represented by photographs of cultivated and spontaneous plants, or for which it was not possible to clearly define the environment, were assigned to both categories “spontaneous” and “cultivated” (S + C).

A further sub-classification was carried out within the “cultivated” category: plants cultivated for their “edible” use, such as the genera *Fragaria* (strawberries) and *Capsicum* (chillies), were distinguished and assigned to the “horticultural” (H) sub-category; likewise, aromatic plants (e.g., *Ocimum,* basil) were assigned the “aromatic” (A) sub-category; all the other ones were considered ornamental (O) plants.

Weighted network representation was chosen to visualize the interactions between bees and plants; in interaction networks, links have width proportional to the importance of the flow. Two different networks have been obtained, considering the spontaneous and the cultivated plants, in order to depict the interactions with these different plant categories. R package networkD3 [24] was used, which also allowed the creation of an interactive version of the two networks.

Statistical analysis was performed by [25]; all other graphs in this publication were constructed using R package ggplot2 [26]. RStudio v.1.3.1073 was used for all elaborations.

## 3. Results

In two years, we received 1086 reports from 269 users. This total does not account for 92 reports, of which 7 were photographs of pinned specimens; 4 did not come from the Italian territory; and 81 were not related to Apoidea but to other insects.

**Figure 2:**
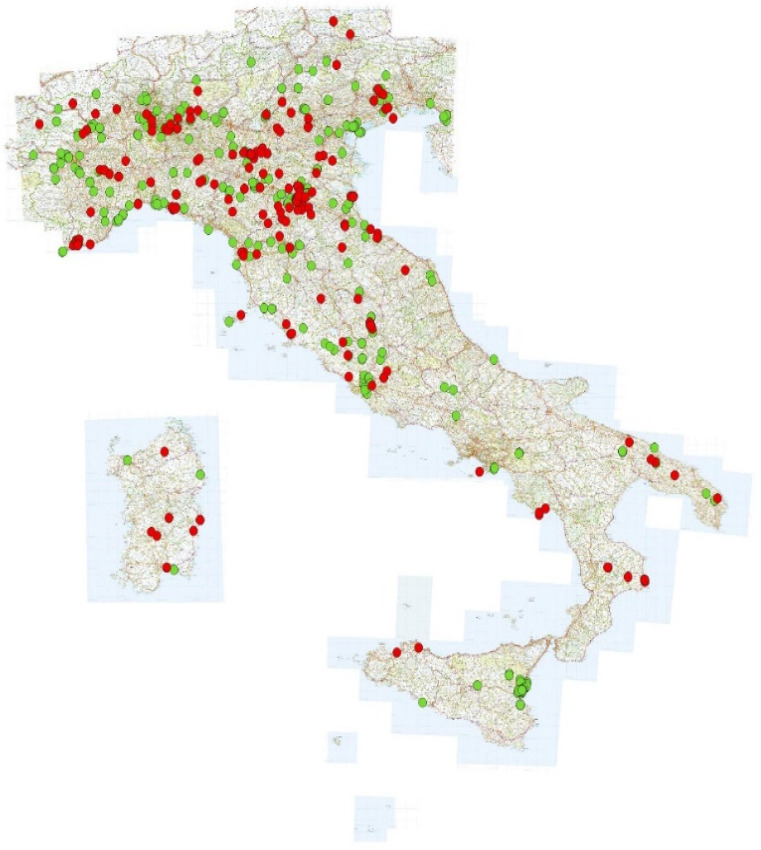
Map of the reports sent in the years 2019 (red dot) and 2020 (green dot).

**Figure 3:**
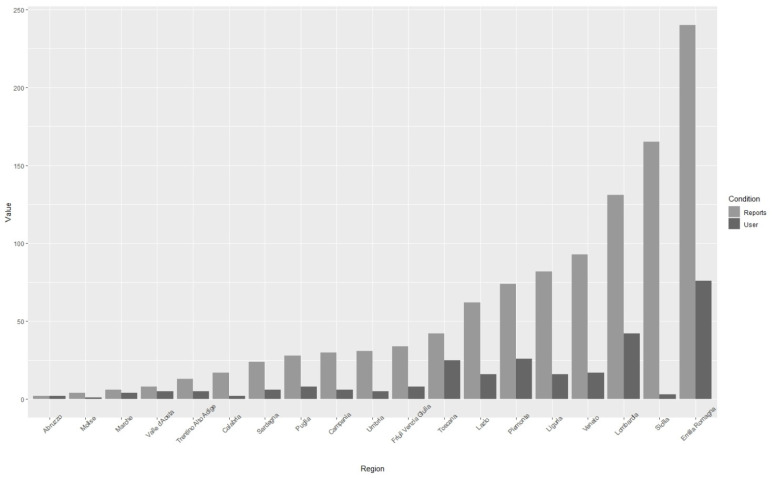
Number of reports and number of users in the different Italian regions.

Figure 2 shows the geographical distribution of reports in Italy for the two years (2019: red dots; 2020: green dots). The reports relating to 2019 amounted to 347 (sent by 115 users), while those for 2020 amounted to 739 (sent by 154 users), with a mean number of reports per user respectively of 3 in 2019 and 4.8 in 2020. In Figure 3 the origin of the reports and users from the different Italian regions is summarized. In some regions, very few users were responsible for most of the reports (e.g., Sicily).

**Figure 4:**
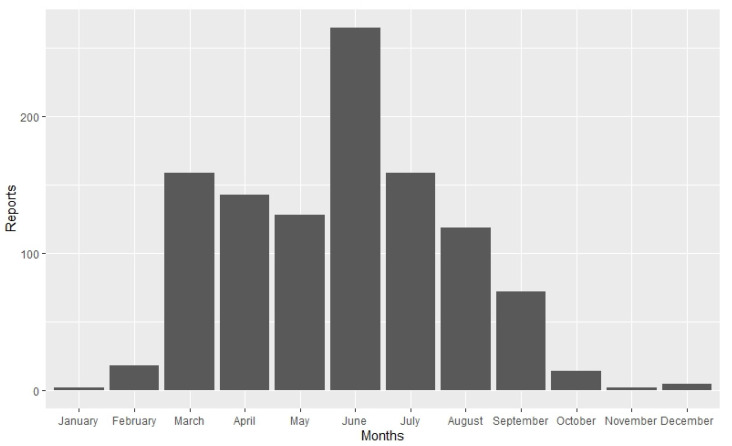
Number of reports along the months in the two years of the project.

Figure 4 shows the number of reports along the months pooling two years of the project. The number of reports reflects the flying season of bees (from March to September in most of the Italian regions), but the very high peak in June is likely due to the beginning of summer vacation in Italy. From June to September the number of reports decreases, in line with the seasonal trend of bee populations.

### 3.1. Bee Reports

Most of the recorded bees were medium sized (510 reports), followed by the large sized (205 reports), small sized (149 reports) and the extra-large ones (111 reports), while the extra-small bees were the least represented (30 reports).

**Figure 5:**
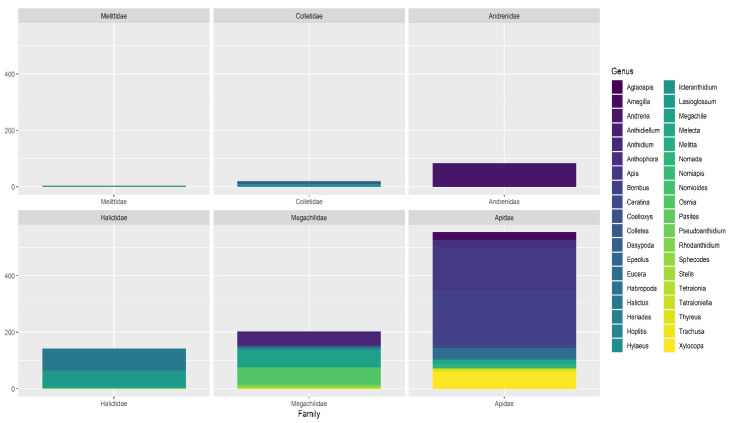
Reported specimens grouped by genus and family.

The Apidae family was the most represented (Figure 5, 554 reports), followed by Megachilidae (202 reports), Halictidae (142 reports), Andrenidae (84), Colletidae (20) and Melittidae (3). A total of 38 genera have been reported (Figure 5 and Table 1); the genera *Bombus* Latreille, 1802 and *Apis* (L., 1768) were the most reported, with more than 150 records, followed by *Andrena* Fabricius, 1775, *Halictus* Latreille, 1804, *Lasioglossum* Curtis, 1833, *Megachile* Latreille, 1802, *Xylocopa* Latreille, 1802 and *Osmia* Panzer, 1806, that obtained between 60 and 84 reports; between 11 and 47 reports were referred to other 8 genera, while 22 different genera obtained have been reported less than 10 times each (Table 1).

The total number of correct reports in the 2 years of activity of the project was 666 (61.3% of the total reports), while the wrong ones were 420 (38.7%). Of the latter, 86 were not related to wild bees but to other insect taxa, while in the others the attribution to the genus was incorrect. The number of correct reports increased from 2019 (57.3%) to 2020 (63.2%), but this difference is not statistically significant ($\chi^{2\;}$, *p* = 0.076).

Table 1 shows the number and percentage of genera correctly and incorrectly reported by the users. All evaluations were made at the genus level, due to the fact that the species rank is very difficult to be defined by non-experts. The last column of the table indicates the percentage of reports for which the project expert taxonomists were able to determine the species through the photos.

Among the genera with more than 10 reports (a lower number do not allow to draw conclusions) the most misreported genus was *Lasioglossum*, with more than 2/3 of incorrect reports; the genera *Apis*, *Andrena*, *Halictus*, *Eucera* Scopoli, 1770, *Anthophora* Latreille, 1803, *Nomada* Scopoli, 1770 and *Ceratina* Latreille, 1802 registered between 1/3 and 2/3 of incorrectness, while the genera *Bombus*, *Megachile*, *Osmia*, *Xylocopa*, *Anthidium* Fabricius, 1805, *Amegilla* Friese, 1897, *Melecta* Latreille, 1802 and *Hylaeus* Fabricius, 1793 were correctly recognised in more than 2/3 of reports. Among “rare” reports, *Hoplitis* Klug, 1807, *Nomiapis* Cockerell, 1919, *Tetraloniella* Ashmead, 1899, *Pseudoanthidium* Friese, 1898, *Trachusa* Panzer, 1804, had 100% misreporting, while *Melitta* Kirby, 1802, *Pasites* June, 1807, *Sphecodes* Latreille, 1804, *Aglaoapis* Cameron, 1901, *Anthidiellum* Cockerell, 1904, on the contrary, had 100% of correct reporting.

Beside the honey bees, the genera for which it was almost always possible to identify the species from the photo by the experts were the big sized or easily recognizable in the picture (e.g., *Bombus*, *Halictus*, *Osmia*, *Megachile, Anthidium*, *Amegilla*), and the ones for which only one or a few species are present in Italy (e.g., *Xylocopa*, *Habropoda*, *Rhodanthidium* Isensee, 1927, *Stelis* Panzer, 1806, *Icteranthidium* Michener, 1948, *Trachusa*, *Aglaoapis*, *Pasites*, *Tetralonia* Spinola, 1839, *Nomiapis*).

### 3.2. Plant Reports and Plant-Pollinator Networks

A total of 190 plant genera were reported in the 2 years, belonging to 59 plant families. Of these, 4 families included more than 10 genera: Compositae with 27 genera representing 14.2% of total reports, Lamiaceae with 17 genera (8.9%), Fabaceae with 16 genera (8.4%) and Rosaceae with 13 genera (6.8%); while 28 families are represented by a single genus.

The majority of reported genera (115) were of spontaneous native flora, while 64 were of cultivated plants. Eleven genera were ascribed to both groups since the reports were referred to both spontaneous and cultivated plants (*Allium*, *Aster*, *Calendula*, *Cyclamen*, *Erica*, *Iris*, *Lavandula*, *Mentha*, *Prunus*, *Rosa* and *Salvia*). Among the cultivated plants, there were 4 genera of typically aromatic plants, usually grown for food purposes (*Lavandula*, *Mentha*, *Ocimum* and *Salvia*), 9 of horticultural plants (*Allium, Capsicum, Citrus, Cucurbita, Foeniculum, Fragaria, Helianthus, Malus, Prunus*) and 62 plant genera grown for ornamental purposes, mainly allochthonous (Supplementary Materials).

**Figure 6:**
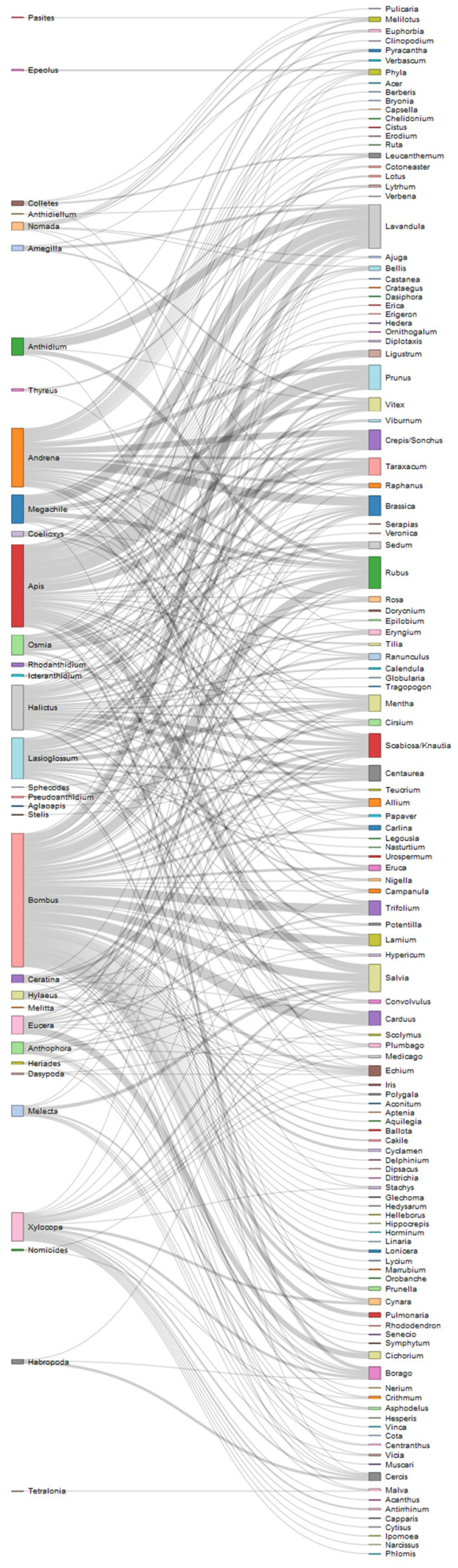
Network of reported interactions between bees and spontaneous plants. Genus taxonomic level is considered.

**Figure 7:**
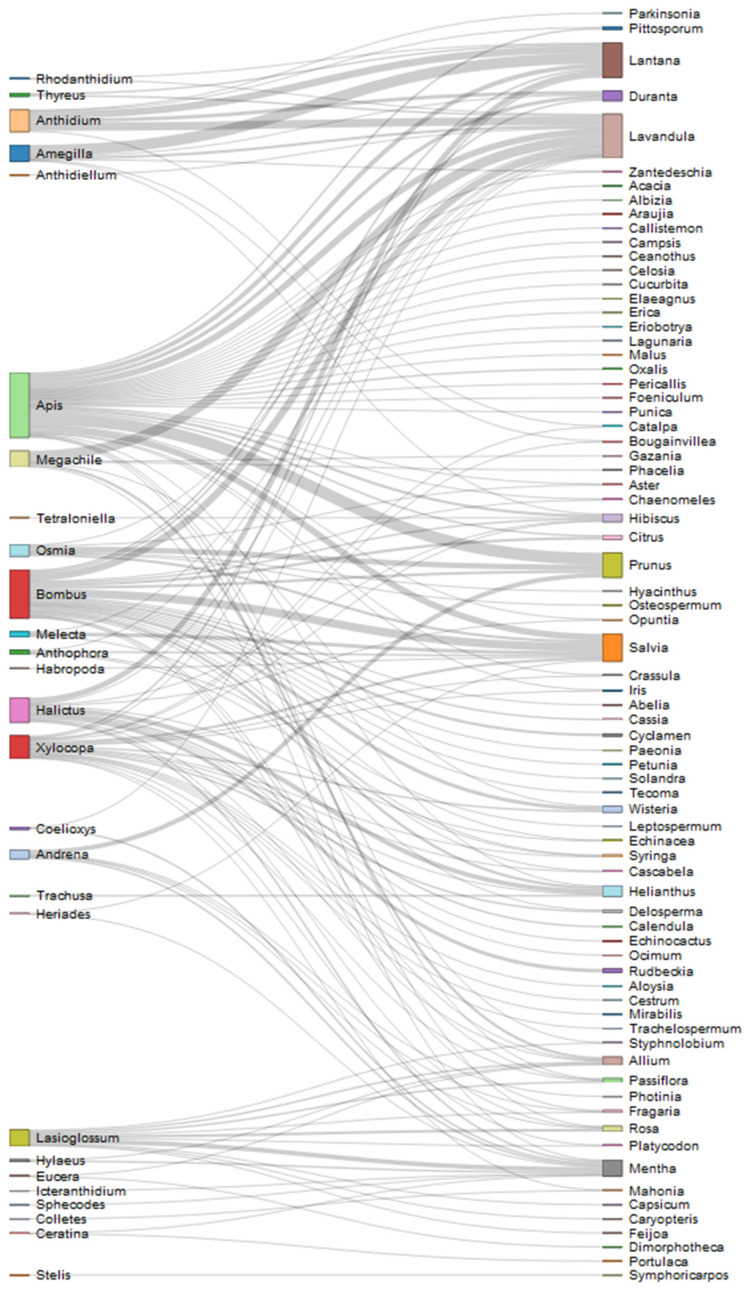
Network of reported interactions between bees and cultivated plants. Genus taxonomic level is considered.

In Figure 6 and Figure 7 plant pollinator networks were designed for both spontaneous and cultivated plants.

Thirty-four genera of bees out of the thirty-eight reported were observed on the flowers of spontaneous plants. The genera *Apis* and *Bombus*, social bees with a flying period extended along the whole season, were seen on flowers of many different genera, although they were more often observed on some of them (e.g., *Apis* on *Prunus*, *Taraxacum*, *Salvia*; *Bombus* on *Lavandula*, *Rubus*, *Trifolium*, *Lamium*, *Salvia* and *Carduus*). Likewise, bees belonging to genus *Halictus*, *Lasioglossum* and *Xylocopa,* which can exhibit a social or pre-social behaviour and a long flying period, have been reported indifferently on several plant genera. In other cases, the observed plant-bee interaction reflects the oligolectic habit of the bee genus (e.g., *Tetralonia* on *Malvae*) or its specialization on specific plants flowering during its flight period (e.g., *Osmia* on *Prunus* trees; Andrena on *Brassica*; *Anthidium* and *Amegilla* on *Lavandula*; etc).

One of the most commonly reported plant genera is *Lavandula*, visited mainly by *Apis*, *Bombus*, *Anthidium* and *Megachile*, followed by *Rubus* (visited by *Bombus*, *Anthidium*, *Megachile, Andrena* and *Halictus* bees), *Mentha* (mainly visited by *Lasioglossum* bees), *Salvia* (with *Apis*, *Bombus*, *Melecta*, *Xylocopa*) and *Carduus*, where almost exclusively bumble bees have been observed.

Interactions with cultivated plants were reported for 21 bee genera out of 38, the most observed being *Apis Bombus Xylocopa*, *Amegilla*, *Halictus*, *Anthidium*. The genus *Apis* has been observed on several different flowers, while *Bombus* mainly on *Salvia*. *Amegilla, Halictus* and *Anthidium* have been reported mostly on *Lantana*, which accounts most of the reports among cultivated plants, although *Halictus* bees were observed almost equally on *Helianthus*. Finally, *Xylocopa* has been observed on several plants with no prevalence.

## 4. Discussion

One of the main purposes of beewatching, as with all citizen science projects, is to enhance citizens’ knowledge, specifically on bee pollinator diversity and their relationship with entomophilous plants [18]. The results obtained in the first two years of the project indicate that the project succeeded in enhancing people knowledge and perception of wild bees and their diversity in Italy. In fact, of the more than a thousand reports received, only a very small fraction did not concern bees, but Diptera or other Hymenoptera.

The distribution of reports, although not homogeneous across the entire territory (there is a clear disparity between Northern and Southern Italy) (Figure 2), includes a good representation of all the Italian provinces. Furthermore, the sharp increase in the number of reports from 2019 to 2020 and the increase in the number of reports from Southern Italy in 2020 highlight a growing reception of the project throughout the Italian peninsula. On the other hand, the objective of obtaining more correct bee identification by the users, through the dissemination work carried out with the web platform and the Facebook page, was not completely achieved, although the results show a positive trend (Table 1)

The fact that medium size and large bees were the most reported was not surprising, since the majority of bee species are out of this size and are easier to note. Conversely, small, and extra-small bees, which are difficult to spot and photograph in the environment, were less reported.

As expected, the most reported bee genera were the very common and widespread *Apis* and *Bombus*, but it is nevertheless interesting to note that some rare and kleptoparasitic genera have been reported (e.g., *Melecta*, *Nomada*, *Thyreus* Panzer, 1806) among which some are very rare (*Aglaoapis*) (Table 1). The kleptoparasitic genera are interesting both since they are generally uncommon [27] and since, being at the apex of the Apoidea community, they can give indications on the general state of bee populations [28]. Moreover, their presence indirectly indicates the presence of their host species.

An important aspect of our project was the possibility to verify the correctness of data sent by participants, named expert-assisted citizen science. With the classical citizen science approach, data are collected and sent directly to scientists, without verification, while with expert-assisted citizen science, people provide samples to expert scientists (taxonomists), who identify specimens to a given taxonomic rank. In our case, both data submission and verification were performed through photographs, since we preferred a non-destructive monitoring of insect pollinators and plants. This choice has reduced the precision at which the data could be analysed (i.e., genus level for identification), still allowing us to obtain results useful for monitoring and to verify the accuracy of the data sent by the participants.

While through other European CS projects that are based on photographic reports and focus on a single easily recognizable genus, e.g., Bombus [14,15], it is possible to reach identification at the species level, our choice to consider all the bee genera and the great biodiversity of bees in Italy did not allow us to identify most of the specimens at the species level. The low number of determinations at the species rank is partly due to the low quality of pictures and the consequent lack of the taxonomic details needed. However, our decision not to require a minimum standard photo quality criterion allowed a wider involvement of citizens.

The bee genera that have been most correctly recognized (Table 1) are those of larger size or with more evident features: the large and hairy bumblebees of genus *Bombus*; the medium sized *Megachile*, with pollen-carrying scopa located on the ventral surface of the abdomen; the very big violet carpenter bee *Xylocopa*; the large carder bee *Anthidium* with its wasp-like appearance; the banded *Amegilla* bee, with fast hovering flight; the large grey-black *Melecta* bees, with slow and gliding flight; and the small and hairless *Hylaeus* bees, called “masked bees” for the typical yellow spots on the head. On the other hand, the most unrecognized genera are those objectively difficult to determine through photos, since they resemble other more common genera, such as the small and not much colourful *Lasioglossum*; the genus *Andrena*, which includes many species of very diversified appearance; the genus *Halictus*, whose species do not show very distinctive traits; and some cryptic and poorly known genera such as *Nomada* and *Ceratina*. At the same time, some uncommon genera have been correctly identified (Table 1), which may reasonably indicate that also expert users participated as beewatchers.

For many genera, the determination of the species by taxonomists was possible in a higher percentage than the correct determination of the genus by the users. Exceptions are represented by some genera that are easy to recognize but difficult to determine at the species level, such as the kleptoparasites *Melecta* and *Nomada* and the yellow masked bees of the genus *Hylaeus*. For some genera, the percentage of correct identification at the genus rank (citizen) and at the species rank (expert) was similar (e.g., *Xylocopa*), probably due to low intrageneric diversity, as for genus *Xylocopa,* which includes a few species, one of which is much more common (*Xylocopa violacea* L., 1758). A similar result was found for the genus *Lasioglossum*, where the taxonomic determination is equally difficult at the level of genus and species.

As for wild bees, identification at species level was not always feasible for plants. Wherever possible, the correct information was provided when the report was approved, while for others only the genus was indicated. The difficulty in plant identification arises from photos rightly focused on the bee and not on the plant visited, so that many identification traits are not distinguishable, or are present on other parts of the plant not framed in the image such as flower sepals and vegetative structures (leaves, bracts, …). Furthermore, the cultivated plants often belong to ornamental cultivars that accentuate some morphological traits compared to the native ones, and it is not always possible to determine the exact name of the species and cultivar. However, users themselves have often suggested the identification of the plants, which is usually accurate at the species level and probably recognized with an id-key or -app, or thanks to personal knowledge, a fundamental help for the experts in the approval phase of the report.

Some CS projects on wild bees do not focus their observations on flowers but on nesting sites, spotting cavity-nesting bees at artificial nest boxes hotels with observations and photography [29]. It was demonstrated that this kind of projects generally obtains a wider participation and best results than those in which the whole biodiversity is addressed, since they limit the bees to be observed to cavity-nesting species [30].

This type of survey can be very useful for monitoring alien bee species, which mainly belong to cavity nesting species, since they greatly reduce observation sites and species to recognize. In this regard, the reports received in the two-year period 2019–2020 (both from the web platform and from the “Beewatching” Facebook page) included 22 reports of *M. sculpturalis*, the main allochthonous specie present in Italy [20]; the other one, *M. disjunctiformis* [21] was not reported. The reports of *M. sculpturalis* received by our web site allow to extend the distribution of the species in Italy [22]; although not numerous, they include reports from central and southern Italy where the species had not been observed before. This result confirms the importance of adopting a citizen science approach for the monitoring of alien species, with lower costs than a data collection carried out directly by research teams [5,31]. The absence of reports for *M. disjunctiformis* may be due to its limited distribution and abundance (which at the moment seems to be confined to the territory of Bologna, Italy) but also due to the small size and inconspicuous appearance.

## 5. Conclusions and Future Perspectives

The first two years of Beewatching project led to some positive results, namely the increasing number of reports and the high percentage of correct ones. However, there are still some improvements that can be made in the next years in order to collect useful information. It might be interesting to outline the profile of “beewatchers” through a questionnaire, in order to plan activities in a more targeted way, but also to start some sub-projects for conservation purposes [18,32]. In fact, apart from knowledge raising, another important output of a citizen science project on pollinators should be the change of attitude of participants towards the studied group. Nature-based citizen science projects can educate their volunteers about conservation issues and encourage their engagement in conservation actions. The study of Lewandowski and Oberhauser [33] showed that volunteers who received information on conservation, and whose engagement was encouraged through the CS project, were more likely to be actively involved in conservation actions. In the case of bees, participants could start to make wild bee nests, following indication on our web site, or to plant melliferous plant taken by the list provided on the web site. During these first two years we did not collect evidence that participants performed these actions, but it would be interesting to verify it in the next years, through a questionnaire aimed not only at understanding how much users became more conscious about the importance and diversity of bees, but also to evaluate their change of behaviour (e.g., what actions they have implemented to favour bee presence and diversity).

## Figures and Tables

**Table 1:** Number (and percentage) of correct and incorrect determination at genus level by the users; in the last column, percentage of records for which species attribution by the experts was possible. (* kleptoparasite genera).

Genera	Total	Correct (by Users)	Incorrect (by Users)	Determination at Species Level (by Experts)
*Bombus*	191	162 (85%)	29 (15%)	98%
*Apis*	155	102 (66%)	53 (34%)	100%
*Andrena*	84	36 (43%)	48 (57%)	56%
*Halictus*	77	38 (49%)	39 (51%)	82%
*Megachile*	61	47 (77%)	14 (23%)	84%
*Lasioglossum*	61	18 (30%)	43 (70%)	28%
*Osmia*	60	40 (67%)	20 (33%)	80%
*Xylocopa*	60	52 (87%)	8 (13%)	87%
*Anthidium*	47	41 (87%)	6 (13%)	98%
*Eucera*	32	19 (59%)	13 (41%)	72%
*Amegilla*	27	22 (81%)	5 (19%)	85%
*Anthophora*	24	14 (58%)	10 (42%)	71%
Melecta *	16	11 (69%)	5 (31%)	50%
Nomada *	14	6 (43%)	8 (57%)	14%
*Ceratina*	11	5 (45%)	6 (55%)	55%
*Hylaeus*	11	8 (73%)	3 (27%)	9%
*Colletes*	9	3 (33%)	6 (67%)	67%
*Habropoda*	8	7 (88%)	1 (13%)	100%
Thyreus *	8	7 (88%)	1 (13%)	63%
*Rhodanthidium*	6	4 (67%)	2 (33%)	83%
Coelioxys *	6	4 (67%)	2 (33%)	33%
*Heriades*	6	4 (67%)	2 (33%)	33%
Stelis *	4	2 (50%)	2 (50%)	100%
*Anthidiellum*	3	3 (100%)	0 (0%)	100%
Epeolus *	3	1 (33%)	2 (67%)	100%
*Icteranthidium*	3	2 (67%)	1 (33%)	67%
*Pseudoanthidium*	3	0 (0%)	3 (100%)	33%
*Nomioides*	2	1 (50%)	1 (50%)	0%
*Dasypoda*	2	1 (50%)	1 (50%)	50%
*Tetraloniella*	2	0 (0%)	2 (100%)	0%
*Trachusa*	2	0 (0%)	2 (100%)	100%
Aglaoapis *	1	1 (100%)	0 (0%)	100%
Pasites *	1	1 (100%)	0 (0%)	100%
Sphecodes *	1	1 (100%)	0 (0%)	0%
*Tetralonia*	1	1 (100%)	0 (0%)	100%
*Hoplitis*	1	0 (0%)	1 (100%)	100%
*Melitta*	1	1 (100%)	0 (0%)	100%
*Nomiapis*	1	0 (0%)	1 (100%)	100%

## Data Availability

Data is contained within the article or supplementary material.

## Supplementary Materials

Supplementary can be found at https://www.mdpi.com/article/10.3390/insects12090841/s1.
